# Profiles of career adaptability and their associations with sociodemographic characteristics, big five traits, and mental health among Chinese primary and secondary school teachers

**DOI:** 10.3389/fpsyg.2026.1841554

**Published:** 2026-06-05

**Authors:** Chunmei She, Longxiang Zou, Jirong Zhou

**Affiliations:** 1 School of Education and Psychological Sciences, Sichuan University of Science and Engineering, Zigong, China; 2 Department of Human Resources, Sichuan University of Science and Engineering, Zigong, China; 3 School of Mechanical Engineering, Sichuan University of Science and Engineering, Zigong, China

**Keywords:** big five traits, career adaptability, career construction theory, latent profile analysis, mental health

## Abstract

In this era of educational and career pathways fragmentation and unpredictability, fostering and sustaining career adaptability has become a major concern. This study contributes to the advancement of research on career adaptability by adopting a person-centered approach and applying the career construction model of adaptation framework in a cross-sectional design on primary and secondary school teachers’ career adaptation in Guangzhou, China (*N* = 1,437). This study aimed to (a) identify distinct profiles of career adaptability based on different combinations of its four facets, (b) examine the associations of profile membership with sociodemographic characteristics, Big Five traits, and mental health. Latent profile analyses revealed a 3-profile solution, namely, relatively lower adaptability, moderate adaptability with stronger control, and high adaptability profiles. The findings showed that most Big Five traits were associated with higher career adaptability profiles, and teachers with non-teaching majors were more likely to belong to the high adaptability profile. Inversely, the agreeableness trait predicted membership only in the moderate adaptability with stronger control profile, rather than in the high adaptability profile. Moreover, differences in mental health were found between the three profiles; teachers with high adaptability profiles showed significantly higher levels of mental health. These findings suggest that level effects are predominant in career adaptability profiles in this Guangzhou teacher sample, suggesting that shared adaptability resources are associated with adaptivity and adaptation.

## 1 Introduction

The teaching career is uniquely characterized by change (Collie and Martin, 2017). Primary and secondary school teachers have to cope with both anticipated changes in their career environment and unexpected changes, such as pedagogical reforms, the impact of artificial intelligence on the traditional role of the teacher, and the crisis of unemployment due to a shrinking student population. Understanding the capacity of teachers to prepare for and cope with these challenges is therefore critical to sustaining and helping them navigate an ever-changing career world. Career adaptability is regarded as an individual’s psychological resource for coping with unknown, complex career tasks and dealing with career setbacks that individual may encounter (Savickas, 2005).

An increasing body of evidence suggests that teachers’ career adaptability is a crucial psychosocial resource for teaching quality, student support (McLean et al., 2023), and resilience in the face of occupational stressors (Kücükaydin, 2023). Teacher career adaptability has also been shown to influence student-level outcomes, such as teacher-student relationships (Fisher et al., 2016) and student academic motivation (Martin et al., 2024). These findings underscore the significance of career adaptability for teachers at both individual and organizational levels, emphasizing its impact on professional development, job performance, and mental health. However, much of the existing research assumes that career adaptability operates uniformly across its dimensions, neglecting the possibility that individuals may exhibit diverse profiles of career adaptability depending on their personal traits, work contexts, and social environments. According to career construction theory, these dimensions interact dynamically within individuals at different levels (Savickas, 2005), and their specific configurations are likely to yield distinct outcomes (Hirschi and Valero, 2015). However, there remains limited research on how these distinct adaptability resources combine within teachers, how such combinations can be inferred from data, whether these profile memberships can be predicted by personality traits (e.g., the Big Five dimensions) and relevant sociodemographic, and the role of these adaptability resources combinations in career adaptation (i.e., mental health). Addressing these gaps is essential for advancing the understanding of the role of career adaptability in educational settings and for tailoring interventions to support teachers’ career development and well-being.

Accordingly, drawing on the career construction model of adaptation framework, the present study identified latent profiles of career adaptability representing distinct configurations of career adaptability among primary and secondary school teachers in Guangzhou, China. Plausible predictors of the probability of profile membership were also examined, including sociodemographic characteristics and career adaptivity (i.e., Big Five traits). In addition, mental health among primary and secondary school teachers in Guangzhou, China was examined was examined as a meaningful career adaptation outcome of career adaptability profile membership. The person-centered investigation of career adaptability profiles in the field of career construction research serves three important roles: (1) to clarify whether career adaptability profiles among teachers are mainly expressed as overall level differences across concern, control, curiosity, and confidence or as more differentiated dimensional patterns. This clarification can refine the theoretical meaning of concern, control, curiosity, and confidence, because these four resources may not operate in the same way across different occupational groups; (2) to advance the explanation of how dispositional and contextual characteristics may jointly relate to teachers’ adaptability profiles. This explanation can move career construction theory beyond a simple main effect account and toward a more conditional understanding of why some teachers are more likely to possess stronger adaptability resources in specific professional contexts; and (3) to broaden the understanding of adaptation outcomes in career construction theory. Teacher mental health can reflect whether teachers have enough psychological resources to respond to work pressure, role demands, and educational change. In addition, the participants in this study are Chinese primary and secondary school teachers, a group that could enrich the occupational and sociocultural diversity of existing person-centered career adaptability research.

## 2 Theory and hypotheses

### 2.1 Career adaptability: theoretical overview

Career adaptability, a key component of career construction theory (CCT), is conceptualized as a multidimensional construct encompassing four distinct yet interrelated dimensions: concern, control, curiosity, and confidence (Savickas and Porfeli, 2012). The aforementioned dimensions collectively constitute the foundation of an individual’s adaptability resources, enabling effective management of career-related tasks, transitions, and challenges (Rudolph et al., 2017).

Consistent with this multidimensional view, career adaptability encompasses both personal judgments of capability and task-specific requirements. Concern refers to future orientation and proactive career planning, while control reflects individuals’ self-discipline and responsibility in shaping their career trajectories. Curiosity is the propensity to explore potential career paths and opportunities, and confidence signifies self-efficacy in overcoming career obstacles (Savickas, 2013). Together, these dimensions form an integrative model that captures the holistic and task-specific nature of adaptability, yet they are not redundant. Empirical evidence indicates that these dimensions share substantial variance and exhibit unique relationships with career-related predictors and outcomes (Rudolph et al., 2017). For instance, curiosity might correlate more strongly with openness to new experiences, and concern is more strongly associated with proactive career management, while confidence may align more closely with resilience in career setbacks, highlighting the need to examine these dimensions both collectively and independently (Rudolph et al., 2017). In addition, during the theoretical conceptualization of career adaptability, researchers have found that developmental trajectories further reveal dimension-specific dynamics. Wang et al. (2024) noted that concern, control, curiosity, and confidence may not develop at the same rates, and that career-related tasks, transitions, and traumas may intervene in the development of these dimensions. As such, factors such as an individual’s personality traits, career tasks, or career stage may interfere with the developmental trajectory of these dimensions, potentially leading to significant intra-individual differences within these dimensions.

Emerging from this theoretical understanding is the recognition that individuals may display differing configurations of career adaptability dimensions, reflecting unique intra-individual patterns. These configurations can be conceptualized through both variable-centered and person-centered approaches. Variable-centered approaches focus on the relationships between adaptability dimensions and career outcomes, whereas person-centered approaches aim to identify distinct adaptability profiles among individuals. Profiles may reveal individuals with uniformly high adaptability across all dimensions or those who are stronger in some areas but weaker in others, illustrating diverse pathways to career adaptability. Although career adaptability is often treated as a higher-order construct in empirical research, studies have also highlighted the importance of accounting for general and specific variance in its dimensions. Failure to disentangle generality from specificity may obscure distinct intra-individual adaptability patterns (Hirschi and Valero, 2015). Thus, the person-centered approach has been proposed to examine configural patterns rather than isolated traits.

### 2.2 Career adaptability: towards a person-centered approach

The theoretical perspective that teachers may not exhibit uniform levels of career adaptability across its dimensions suggests the existence of distinct unobserved subgroups with unique combinations of adaptability resources. However, this assumed heterogeneity in career adaptability among teachers has been largely neglected in prior research. Existing studies predominantly employ variable-centered approaches to examine career adaptability, focusing on its dimensions as independent predictors of career outcomes (Collie et al., 2020; McLean et al., 2023). While this approach has proven pivotal in shaping the nomological framework of career adaptability and clarifying its mechanisms in promoting career success and well-being, it assumes a uniform adaptability growth in the sample population, thereby neglecting potential unobserved subpopulations with distinct profiles of career adaptability.

A person-centered approach, which emphasizes multidimensional configurations of adaptability resources, offers a novel lens to explore the interplay among career adaptability dimensions. One such attempt was made in Hirschi and Valero’s (2015) research on the career adaptability of German university students, in which five profiles were identified across two independent samples. Comparisons among these profiles indicated that students with higher adaptability profiles reported significantly higher adapting and adaptivity. Building upon this, recent studies have expanded this approach to explore more complex dynamics across different populations. For instance, Ojala et al. (2023) identified four career adaptability profiles among Finland student-athletes. Those with higher career adaptability were less likely to withdraw from competitive sports and had higher grade point averages compared to those with lower career adaptability profiles. Similarly, Gong et al. (2023) identified four profiles among Chinese employees, with the optimal and high career adaptability profiles demonstrating high levels of psychological safety and self-efficacy.

From these theoretical standpoints, adaptability resources interact dynamically, and their combined effects likely shape how teachers navigate career challenges and development in the unique sociocultural context of China, where Confucian values emphasize stability, effort, and relational harmony. For example, teachers in this context may exhibit profiles with elevated concern and control dimensions, reflecting a strong sense of responsibility and long-term planning. However, low levels of curiosity may reflect cultural tendencies to prioritize established norms over the exploration of new career paths. The application of a person-centered approach enables the identification of quantitatively and qualitatively distinct profiles among Chinese primary and secondary school teachers. Quantitative differences may manifest in profiles with uniformly high or low levels across all career adaptability dimensions, representing broad variations in overall adaptability capacity. Conversely, qualitative distinctions may manifest as profiles with specific strengths and weaknesses in adaptability dimensions (Morin et al., 2018). For instance, one profile might exhibit high levels of concern and control but low levels of curiosity and confidence, reflecting a structured yet risk-averse approach to career management. Such nuanced profiles highlight the complex interplay between adaptability resources and underscore the limitations of variable-centered methods in capturing these patterns. Accordingly, in line with the person-centered perspective, this study expects the following result:

*Hypothesis 1:* Career adaptability emerges in distinct profiles, which vary both quantitatively and qualitatively.

### 2.3 Antecedents of career adaptability profile membership

A critical challenge for advancing career construction theory is understanding how multidimensional configurations of career adaptability resources emerge from individual differences in sociodemographic characteristics and personality traits. Sociodemographic factors, including age, teaching experience, gender, academic degree, professional attributes, and job title, are hypothesized to influence career adaptability profiles. Age and years of teaching experience, in particular, may affect how teachers develop and deploy their adaptability resources over time. Drawing from the career construction theory, early-career teachers may prioritize concern and curiosity as they explore professional opportunities and anticipate future challenges, while mid- and late-career teachers may focus on control and confidence as they consolidate their career trajectories. Zacher’s (2014) study provides evidence supporting this view, demonstrating that factors such as age and education predicted changes in one or more of the four dimensions of career adaptability over time. This variability underscores the potential existence of distinct career adaptability profiles tied to age factor.

Gender differences may also inform adaptability profiles through socialization processes that shape individuals’ experiences in the workplace. Social cognitive perspectives suggest that gender roles and societal expectations influence the development and expression of career-related resources (Lent and Brown, 2013). In the Chinese context, female teachers represent the largest proportion of the teaching profession, and they may exhibit higher concern and agreeableness due to cultural expectations of nurturance and relational harmony. In contrast, male teachers, who are a minority in the profession, may demonstrate elevated confidence and control, reflecting the need to assert competence in a female-dominated environment. Finally, academic qualifications, professional attributes, and job titles may serve as indicators of opportunities for career growth and resource accumulation, shaping teachers’ career adaptability profiles. For example, teachers with advanced degrees or senior professional titles may have greater access to professional development opportunities, which foster adaptability through mastery experiences and exposure to novel career challenges (Savickas and Porfeli, 2012). These experiences likely enhance control, confidence, and curiosity, contributing to distinctive adaptability profiles among highly qualified teachers. Therefore, we could expect that higher education would protect individuals from precarity and career unsustainability.

According to career construction theory, career adaptability develops as individuals interact with their environments, drawing on psychosocial resources to manage career-related transitions and challenges (Savickas, 2013). Previous research demonstrates robust links between personality traits (such as Big Five personality traits) and career adaptability (Rudolph et al., 2017). Consistent with this view, Perera and McIlveen (2017) utilized trait interactions, specifically Big Five personality profile membership, to represent career adaptivity. They examined the associations between adaptivity profile membership and various outcomes, such as career adaptability (adaptability), organized study behaviors (adapting), and career-choice satisfaction (adaptation). The results indicated that the latent profiles differ in terms of career adaptability levels. Considering the aforementioned observations, two hypotheses are consequently formulated as follows.

*Hypothesis 2:* Being male, older, with a teacher training major, high education, and title would increase the probability of falling into the high career adaptability profile, whereas being female, younger, with a non-teacher training major, lower education and title would increase the probability of falling into the lower career adaptability profile.

*Hypothesis 3:* Low levels of negative emotionality and high levels of the four other traits will increase the probability of falling into high career adaptability profiles.

### 2.4 Outcomes of profile membership

Mental health refers to a positive state of psychological functioning and the absence of significant distress (Barry, 2012). CCT has provided a robust frame for how career adaptability can facilitate mental health (Rudolph et al., 2017). Research indicates that career adaptability supports mental health by enhancing individuals’ sense of agency and reducing the psychological toll of uncertainty and change (Shava and Chinyamurindi, 2021). Teachers who possess high career adaptability are more capable of handling occupational stressors, engaging in proactive career planning, and maintaining mental health (McLean et al., 2023). However, previous research has predominantly examined career adaptability through a variable-centered perspective, concentrating on the independent effects of its dimensions on outcomes. This approach has limited our understanding of how distinct intraindividual patterns of career adaptability resources influence mental health outcomes.

Theoretical and empirical evidence suggests that individuals are likely to possess varying levels of career adaptability dimensions, resulting in heterogeneous profiles (Bouckenooghe et al., 2022). The present study expects distinct configurations of career adaptability resources may exert differential impacts on mental health. Prior research, for instance, has revealed that employees with high levels of confidence and control may excel in strategic decision-making and task execution, but if coupled with low levels of curiosity and concern, they may struggle with implementing their plans effectively, potentially leading to increased stress and reduced psychological safety (Gong et al., 2023). Based on these findings, we believe that teachers with high levels of all facets are more likely to display higher levels of mental health because they can regulate their psychological resources to cope with external pressures and keep physical and mental balance in their working environment. Parmentier et al. (2022) research supports this position, stating that the high profile, characterized by the highest levels across all dimensions, is positively correlated with university students’ career transitions, as it is associated with a sense of positive anticipatory emotions and career decision-making self-efficacy. In accordance with these findings, this study expects the following:

*Hypothesis 4:* Career adaptability profiles will display significant differences in terms of teacher mental health.

## 3 Materials and methods

### 3.1 Participants

A questionnaire survey was conducted using a convenience sampling method among 1,544 primary and secondary school teachers in Guangzhou, China. After excluding invalid responses, a total of 1,437 valid questionnaires were retained. The final sample comprised 1,149 females (80%) and 288 males (20%), with ages ranging from 20 to 44 years (M = 26.33, SD = 3.69). Within the sample, 60.6% of public-funded normal students (*N* = 871), participants were enrolled in a range of professional titles, including intermediate and above titles in primary and secondary schools (4.7%), level 2 titles (26.3%), and ungraded titles (69.0%). For degrees, 65.3% had bachelor’s degrees (*N* = 939), and 34.7% had master’s degrees (*N* = 498).

### 3.2 Measures

#### 3.2.1 Career adaptability

Career adaptability was assessed using the 24-item Career Adapt-Abilities Scale for China (Hou et al., 2012). This scale evaluates four dimensions: concern, control, curiosity, and confidence, with each dimension comprising six items. Participants rated each item on a 5-point Likert scale (1 = strongly disagree, 5 = strongly agree), with higher scores indicating greater perceived career adaptability. In the present study, the Cronbach’s *α* coefficients were 0.87 for concern, 0.86 for control, 0.87 for curiosity, 0.89 for confidence, and 0.96 for the overall scale.

#### 3.2.2 Mental health

To assess the mental health status of Chinese teachers, we employed the unidimensional 12-item General Health Questionnaire (Lai and Yue, 2000). This instrument evaluates psychological distress and well-being through self-reported responses to items such as “Feeling like a worthless person.” Participants rated each item using a four-point Likert scale ranging from 0 (not at all) to 3 (much more than usual). All items were reverse-coded such that higher total scores indicated more favorable mental health conditions. In the present study, the Cronbach’s α coefficient was 0.93.

#### 3.2.3 The big five inventory

We used a 60-item Big Five Inventory-2 (Zhang et al., 2022) to assess the personality traits based on the five-factor model: negative emotionality, extraversion, openness-mindedness, agreeableness, and conscientiousness. Each dimension was evaluated using 12 items, rated on a five-point Likert scale ranging from 1 (strongly disagree) to 5 (strongly agree). The negative emotionality dimension was reverse-scored before analysis, such that higher scores indicate lower negative emotionality and greater emotional stability. In the present study, the reliabilities were 0.92 for extraversion, 0.95 for agreeableness, 0.92 for conscientiousness, 0.93 for negative emotionality, 0.95 for open-mindedness, and 0.97 for the full scale.

### 3.3 Procedure and data analysis

Data for this study were gathered through WJX’s online survey platform. IBM SPSS 23 was utilized to perform the preliminary analyses. To examine the potential presence of common method bias, a confirmatory factor analysis (CFA) comparing a single-factor model (all items loading on one factor) with the theoretical seven-factor model (career adaptability, mental health, and the Big Five dimensions) was conducted (Podsakoff et al., 2003). The single-factor model exhibited poor fit: *CFI* = 0.52, *RMSEA* = 0.07, *SRMR* = 0.09. In contrast, the seven-factor model demonstrated acceptable fit: *CFI* = 0.89, *RMSEA* = 0.04, *SRMR* = 0.04. The substantial improvement in fit (*ΔCFI* = 0.38) suggests that common method bias is unlikely to seriously distort the substantive conclusions. Mplus7.0 was employed to conduct latent profile analysis (LPA). To assess the adequacy of fit of the model, we selected the following less conservative recommended fit values of Hu and Bentler (1999): *CFI* > 0.90, *TLI* > 0.90, *RMSEA* < 0.10, AIC (smaller the better), and SRMR < 0.08, 1 < CHI-SQR/DF < 5. The ideal profile solution should exhibit lower values for AIC, BIC, and aBIC, an entropy greater than 0.80, and significant aLMR and BLRT statistics (*p* < 0.05) (Nylund et al., 2007).

## 4 Results

### 4.1 Preliminary analyses

The results of the means, standard deviations, and correlations of the variables are shown in Table 1: Primary and secondary teachers’ career adaptability scores were highest on the career control dimension and lowest on the career curiosity dimension; all dimensions of primary and secondary teachers’ career adaptability scores were significantly positively correlated with mental health (*p* < 0.01). The CFA results for all measurement models for career adaptability (*χ*^2^/df = 4.17; TLI = 0.96; *CFI* = 0.96; *SRMR* = 0.03; and *RMSEA* = 0.05) and Five traits (*χ*^2^/df = 4.53; *TLI* = 0.90; *CFI* = 0.90; *SRMR* = 0.04; and *RMSEA* = 0.05) indicated acceptable fit with the data. For mental health, the fit indices were *χ*^2^/df = 14.88, *TLI* = 0.91, *CFI* = 0.92, *SRMR* = 0.04, and *RMSEA* = 0.10. Although the *χ*^2^/df value exceeded the recommended range, this index is sensitive to sample size and should be interpreted together with other fit indices. The RMSEA value was at the upper bound of acceptability, while the CFI, TLI, and SRMR values met the adopted criteria. Taken together, the mental health measurement showed mixed but generally acceptable fit with the data. These findings further supported the adequacy of the measurement models’ internal validity.

**Table 1:** Means, standard deviations, and correlations of the variables.

Variables	Mean	SD	1	2	3	4	5	6	7	8	9
1. Concern	4.26	0.42	–								
2. Control	4.37	0.44	0.69^**^	–							
3. Curiosity	4.23	0.47	0.74^**^	0.73^**^	–						
4. Confident	4.30	0.46	0.74^**^	0.73^**^	0.80^**^	–					
5. EX	4.25	0.50	0.51^**^	0.48^**^	0.49^**^	0.56^**^	–				
6. AG	4.48	0.47	0.36^**^	0.40^**^	0.36^**^	0.41^**^	0.41^**^	-			
7. CO	4.46	0.42	0.55^**^	0.55^**^	0.54^**^	0.63^**^	0.62^**^	0.54^**^	-		
8. NE	4.34	0.46	0.42^**^	0.44^**^	0.43^**^	0.52^**^	0.58^**^	0.42^**^	0.64^**^	-	
9. OM	4.15	0.56	0.48^**^	0.46^**^	0.52^**^	0.53^**^	0.53^**^	0.38^**^	0.57^**^	0.46^**^	-
10. MH	3.51	0.39	0.40^**^	0.44^**^	0.44^**^	0.44^**^	0.36^**^	0.29^**^	0.46^**^	0.34^**^	0.36^**^

EX, Extraversion; AG, Agreeableness; CO, Conscientiousness; NE, Negative Emotionality; OM, Open-Mindedness; MH = Mental Health; **p < 0.01.

### 4.2 Latent profile analysis

**Figure 1:**
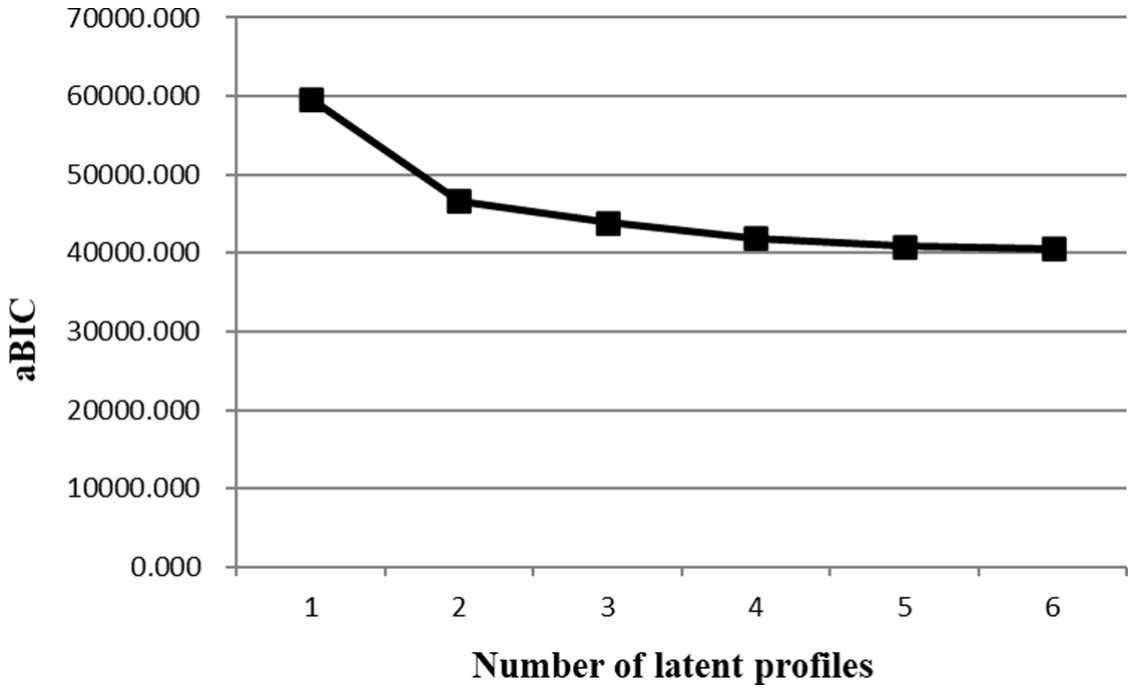
Steep slope plot of aBIC values.

Based on the 24 first-order dimension scores from the career adaptability scale, one to six latent profile models were fitted separately, starting with a solution of one profile baseline model and adding one profile at a time. The results of the latent profile analysis of primary and secondary teachers’ career adaptability are shown in Table 2. The entropy values of all models were greater than 0.90. The LMR values supported the two- and three- profile solutions, and the BLRT results supported the two- to six- profile solutions. The AIC, BIC, and aBIC values decreased monotonically with the addition of profiles. This pattern indicated that models with more profiles provided better statistical fit. However, information criteria were not used as the only basis for profile selection. In practice, concerns regarding parsimony, theoretical adequacy, practical meaning, profile redundancy, and profile size are combined to determine the profile enumeration process, in addition to the availability of fit statistics (Nylund et al., 2007). Figure 1 shows the changes in aBIC values across the one- to six- profile models. The aBIC values continued to decrease as the number of profiles increased. However, the additional decrease became smaller after the three- profile model. This pattern suggested diminishing incremental improvement in model fit after the three- profile solution. Although the four, five, and six profile models showed lower information criteria than the three profile model, these more complex solutions did not provide sufficient added value. The four-profile solution produced one small profile of 4.50%, and the best loglikelihood value was not replicated. The six-profile solution also failed to replicate the best loglikelihood value, and three perturbed starting value runs did not converge. Although the five-profile solution replicated the best loglikelihood value, it produced a warning about a non-positive definite first order derivative product matrix. These results reduced confidence in the stability of the more complex solutions. In addition, the four, five, and six profile models mainly divided the sample into smaller subgroups without providing clearer theoretical meaning. Therefore, the three-profile model was retained as the final model.

**Table 2:** Model fit indices for latent profile analysis.

Model	*k*	AIC	BIC	aBIC	Entropy	LMR(*p*)	BLRT(*p*)	Class probability
1	48	59586.69	59839.67	59687.19	–	–	–	–
2	73	46525.58	46910.31	46678.42	0.96	0.0000	0.0000	0.61/0.39
3	98	43666.72	44183.21	43871.90	0.93	0.0000	0.0000	0.39/0.34/0.27
4	123	41649.31	42297.56	41906.83	0.98	0.4011	0.0000	0.05/0.30/0.28/0.37
5	148	40566.68	41346.69	40876.54	0.96	0.1103	0.0000	0.05/0.37/0.21/0.18/0.19
6	173	40166.21	41077.97	40528.41	0.95	0.7590	0.0000	0.05/0.37/0.14/0.18/0.09/0.17

K, number of freely estimated parameters, AIC, Akaike information criteria, aBIC = adjusted Bayesian Information Criterion, Entropy, Classification accuracy index, BLRT, Bootstrap Likelihood Ratio Test, LMR, Lo Mandell Rubin Likelihood Ratio Test.

**Figure 2:**
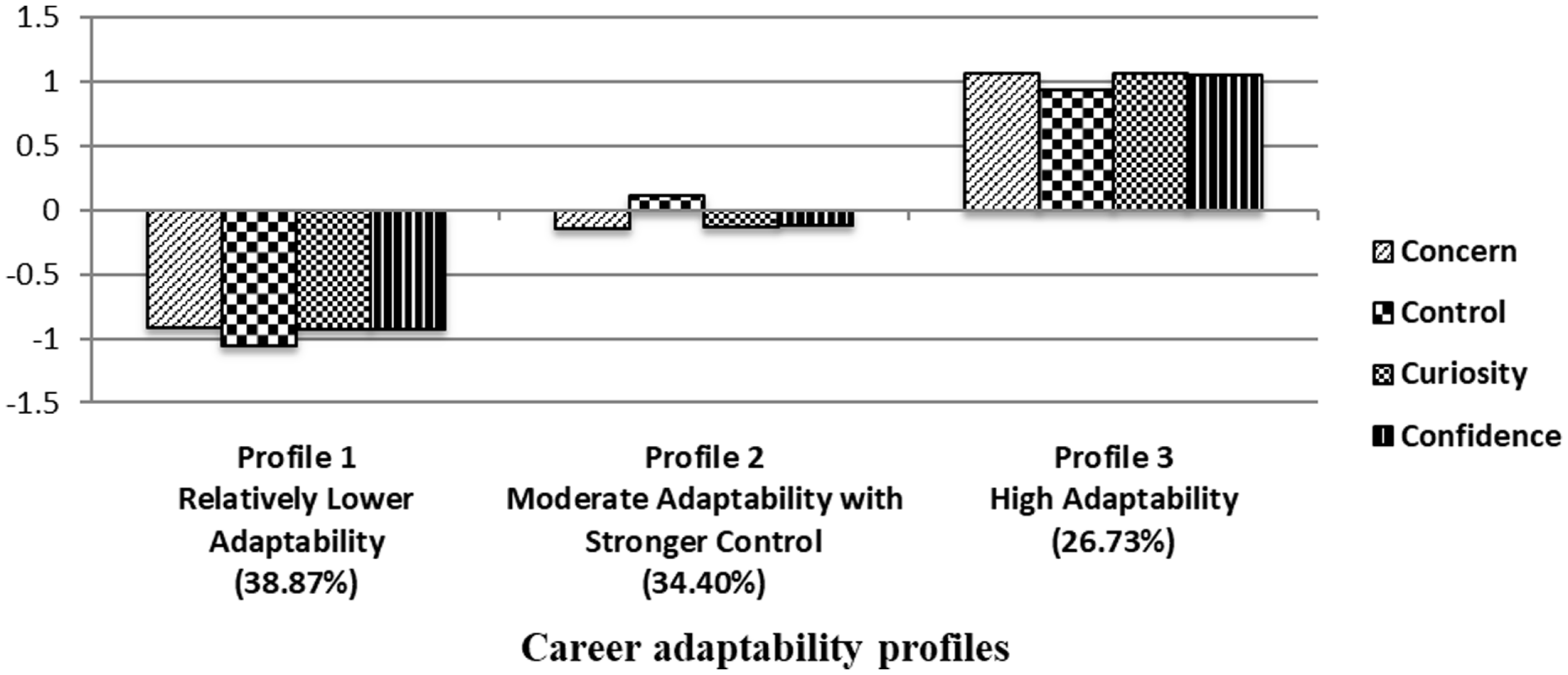
Characteristics of final three-profile solution of career adaptability.

Based on the standardized mean scores of the above latent profile analysis, each profile was interpreted and named (Figure 2). The first profile, representing 38.87% of the cases, exhibited the lowest scores across the four career adaptability dimensions relative to the other two profiles. However, its mean scores were still above the midpoint of the five-point scale. This profile was therefore labelled relatively lower adaptability. The second profile, comprising 34.40% of the cases, displayed moderate scores across the four dimensions, with a relatively higher score on control. This profile was therefore labelled moderate adaptability with stronger control. The third profile (26.73%) showed the highest levels across all four adaptability dimensions and was labelled high adaptability.

Table 3 presents the raw means and standard errors of the four career adaptability dimensions across the three profiles. The relatively lower adaptability profile had the lowest mean scores on concern, control, curiosity, and confidence. The high adaptability profile had the highest mean scores on all four dimensions. The moderate adaptability with stronger control profile was located between these two profiles, and its control score was higher than its concern, curiosity, and confidence scores. These results indicated that the three profiles mainly reflected relative differences in the overall level of career adaptability. Given the high correlations among the four career adaptability dimensions, this level-based pattern should be interpreted cautiously. It may reflect both substantive differences in teachers’ overall adaptability resources and shared variance among the CAAS dimensions. Therefore, Hypothesis 1 was partially supported. The results supported the existence of distinct career adaptability profiles, but they provided stronger evidence for level-based differences than for strong qualitative differences.

**Table 3:** Means and standard errors of career adaptability profiles.

Variables	Mean (S. E.)		
	Profile 1	Profile 2	Profile 3
Concern	3.92 (0.02)	4.25 (0.12)	4.76 (0.11)
Control	3.95 (0.03)	4.47 (0.18)	4.84 (0.07)
Curiosity	3.84 (0.02)	4.22 (0.07)	4.80 (0.07)
Confidence	3.90 (0.04)	4.30 (0.15)	4.88 (0.05)

### 4.3 Predictors of career adaptability profiles

In order to explore the characteristics of different demographic variables and personality traits of career adaptability profile groups, three profiles of teachers’ career adaptability were used as dependent variables, with the relatively lower adaptability as the reference group. The demographic variables included gender, age, years of teaching experience, title, professional attributes, education level and the Big Five traits were taken as independent variables. As shown in Table 4, only gender and professional attributes were predictors for the moderate adaptability with stronger control and high adaptability groups, respectively. Specifically, the regression coefficient of gender on the moderate adaptability with stronger control (*B* = −0.69, *p* < 0.001, *OR* = 0.50) indicated that female teachers had a lower probability of belonging to the moderate adaptability with stronger control profile as compared to their male counterparts. The regression coefficient of professional attributes on the high adaptability (*B* = 0.39, *p* < 0.05, *OR* = 1.48) indicated that the probability of belonging to the high adaptability for non-teaching major teachers was 1.48 times higher than that of normal teachers. Thus, from a gender perspective, male teachers had a higher probability of belonging to the moderate adaptability with stronger control, while female teachers had a higher probability of belonging to the relatively lower adaptability. From the perspective of professional attributes, non-teaching major teachers have a higher probability of belonging to the high adaptability, while normal teachers have a higher probability of belonging to the relatively lower adaptability. Overall, these results indicated that differences in demographic variables (gender and attribution) are important for explaining membership in career adaptability profile groups. In summary, Hypothesis 2 was partially supported.

**Table 4:** Demographic characteristics in relation to different career adaptability profiles.

PV	Moderate adaptability with stronger control			High adaptability		
	*B*	*SE*	*OR*	*B*	*SE*	*OR*
Age	0.01	0.04	1.01	0.02	0.05	1.02
Teaching Year	0.00	0.05	1.00	−0.04	0.08	0.96
Gender (Male = 0)	−0.69^***^	0.20	0.50	−0.38	0.26	0.68
Academic Degree (Bachelor = 0)	0.16	0.20	1.17	0.08	0.26	1.08
PA (Normal Teachers = 0)	0.01	0.15	1.01	0.39^*^	0.20	1.48
Title (intermediate and above = 0)						
Level 2	−0.12	0.41	0.89	0.83	0.55	2.29
Ungraded	−0.46	0.50	0.63	0.12	0.65	1.13
Extraversion	0.40^*^	0.21	1.49	1.39^***^	0.28	4.00
Agreeableness	0.53^**^	0.18	1.69	0.43	0.25	1.53
Conscientiousness	1.55^***^	0.27	4.69	3.39^***^	0.44	29.52
Negative Emotionality	0.17	0.21	1.18	0.78^**^	0.28	2.17
Open-Mindedness	0.54^**^	0.19	1.71	1.48^***^	0.26	4.41

Reference Group, relatively lower adaptability, OR, Odds ratios, PA, professional Attributes, PV, predictor variable; negative emotionality was reverse-scored, with higher scores indicating lower negative emotionality and greater emotional stability. *p < 0.05, **p < 0.01, ***p < 0.001.

Overall, higher levels of Big Five traits were consistently linked to profiles that exhibited higher levels across all facets of career adaptability. High levels of extraversion (*B* = 0.40, *p* < 0.05, *OR* = 1.49; *B* = 1.39, *p* < 0.001, *OR* = 4.00, for the moderate adaptability with stronger control and the high adaptability profiles, respectively), conscientiousness (*B* = 1.55, *p* < 0.001, *OR* = 4.69; *B* = 3.39, *p* < 0.001, *OR* = 29.52, for the moderate adaptability with stronger control and the high adaptability profiles, respectively), and open-mindedness (*B* = 0.54, *p* < 0.01, *OR* = 1.71; *B* = 1.48, *p* < 0.001, *OR* = 4.41, for the moderate adaptability with stronger control and the high adaptability profiles, respectively) were linked to a greater likelihood of belonging to the moderate adaptability with stronger control and high adaptability profiles, as compared to the relatively lower adaptability profile. These mean that extraversion, conscientiousness, and open-mindedness increased the probability of being classified in the moderate adaptability with stronger control and high adaptability profiles. Under the influence of agreeableness, the probability of belonging to the moderate adaptability with stronger control profile was significantly higher (*B* = 0.53, *p* < 0.01, *OR* = 1.69) than that of belonging to the relatively lower adaptability group. This means that agreeableness increased the likelihood of being classified into the moderate adaptability with stronger control profile rather than the relatively lower adaptability profile. However, agreeableness did not significantly predict membership in the high adaptability profile. In addition, low levels of negative emotionality were linked to a higher likelihood of being classified into the high adaptability profile as opposed to the relatively lower adaptability (*B* = 0.78, *p* < 0.01, *OR* = 2.17). This means that low negative emotionality increases the probability of being classified in the high adaptability profile. The findings indicated that differences in personality traits are crucial for explaining membership in career adaptability profile groups. In summary, Hypothesis 3 was partially supported.

### 4.4 Outcomes of career adaptability profiles

As shown in Table 5, according to the results of mean levels across career adaptability profiles for mental health variable and comparisons overall and between-profile means, significant differences between career adaptability profiles could be found for the total mean score of mental health as well as the scores of each item (*p* < 0.001). Overall, profiles exhibiting higher levels of career adaptability across all facets were associated with higher levels of mental health. The high adaptability profile group had the highest mean scores on each item of mental health as well as the total mean score, followed by the moderate adaptability with stronger control group, the relatively lower adaptability group, which had the lowest mental health level score. These results supported the fourth hypothesis and indicated that the different career adaptability profiles also differ in terms of their mental health.

**Table 5:** Relationship between career adaptability profiles and GHQ.

DV	Mean (S.E.)			BCH*χ*^2^			
	Profile1 (*n* = 559)	Profile2 (*n* = 496)	Profile3 (*n* = 382)	Overall test	P1 vs. P2	P1 vs. P3	P2 vs. P3
GHQ1	3.30 (0.02)	3.51 (0.03)	3.72 (0.02)	165.62^***^	36.11^***^	165.33^***^	34.35^***^
GHQ2	3.31 (0.02)	3.57 (0.02)	3.78 (0.02)	241.51^***^	66.03^***^	240.43^***^	40.86^***^
GHQ3	3.33 (0.02)	3.66 (0.02)	3.81 (0.02)	274.78^***^	107.03^***^	267.49^***^	24.86^***^
GHQ4	3.32 (0.02)	3.53 (0.03)	3.73 (0.02)	161.43^***^	39.89^***^	160.20^***^	30.67^***^
GHQ5	3.31 (0.02)	3.57 (0.03)	3.73 (0.02)	175.41^***^	61.45^***^	169.50^***^	20.19^***^
GHQ6	3.32 (0.02)	3.52 (0.02)	3.79 (0.02)	253.16^***^	41.66^***^	252.47^***^	66.98^***^
GHQ7	3.28 (0.02)	3.46 (0.03)	3.73 (0.02)	189.48^***^	28.60^***^	188.88^***^	55.46^***^
GHQ8	3.29 (0.02)	3.52 (0.02)	3.79 (0.02)	281.73^***^	49.17^***^	281.48^***^	69.28^***^
GHQ9	3.37 (0.02)	3.57 (0.02)	3.80 (0.02)	197.50^***^	35.10^***^	196.89^***^	50.01^***^
GHQ10	3.30 (0.02)	3.54 (0.02)	3.79 (0.02)	261.66^***^	54.33^***^	261.66^***^	55.50^***^
GHQ11	3.26 (0.02)	3.50 (0.03)	3.75 (0.02)	224.80^***^	45.62^***^	224.72^***^	47.79^***^
GHQ12	3.31 (0.02)	3.50 (0.02)	3.81 (0.02)	287.23^***^	37.21^***^	283.65^***^	87.41^***^
GHQT	3.31 (0.02)	3.54 (0.02)	3.77 (0.02)	428.51^***^	97.45^***^	428.51^***^	96.69^***^

Profile1, Relatively Lower Adaptability, Profile2, Moderate Adaptability with Stronger Control, Profile3, High Adaptability; P1, Profile 1, P2, Profile 2, P3, Profile 3; DV, Dependent Variables; ***p < 0.001.

## 5 Discussion

This study explored career adaptability profiles using a person-centered approach and investigated their relations with demographic variables and Big Five traits. Further, we identified characteristics of mental health among Chinese primary and secondary school teachers with different latent profiles. Given the exploratory and inductive characteristics of person-centered approaches, such investigations are critical for determining the practical utility of these profiles for professional practice and intervention strategies.

### 5.1 Latent profiles of career adaptability

The LPA results yielded three well-distinguished and meaningful career adaptability profiles. The results suggested that the career adaptability of primary and secondary school teachers could be distinguished into three subgroups: relatively lower adaptability, moderate adaptability with stronger control, and high adaptability, which indicated that there are individual differences within them. The relatively lower adaptability profile represented 38.87% of the sample and described teachers who displayed the lowest scores across all four dimensions relative to the other two profiles. However, this profile should not be interpreted as an absolutely deficient group because its mean scores were still above the midpoint of the five-point scale. The moderate adaptability with stronger control profile represented 34.40% of the sample. This profile displayed moderate levels across the four dimensions, with a relatively higher level of control. The high adaptability profile represented 26.73% of the sample and encompassed primary and secondary school teachers who had the highest levels on all dimensions. The first two profiles together accounted for 73.27% of the sample. This is in line with the Opinions on Comprehensively Deepening the Reform of Teacher Team Construction in the New Era, issued in January 2018, which points out that “in the face of the new orientation, a new journey and a new mission, the construction of the teacher team is not yet fully adapted. Some teachers’ quality and ability are difficult to adapt to the needs of talent cultivation in the new era” (Central Committee of the Communist Party of China, and State Council of the People’s Republic of China, 2018).

### 5.2 Predictors of latent profiles

#### 5.2.1 The role of sociodemographic characteristics in career adaptability profiles

This study provided further insight into how sociodemographic variables may be associated with career adaptability profiles. Consistent with Hypothesis 2, being a female teacher was associated with a lower likelihood of belonging to the moderate adaptability with stronger control profile relative to the relatively lower adaptability profile. This indicates that gender-related career pressures may be associated with relatively lower career adaptability among female teachers. This may be attributable to a confluence of societal expectations, workplace stressors, and personal responsibilities, which disproportionately impact women in the teaching profession. Previous studies have indicated that female educators may experience some unique pressures, such as work-family balance, and these pressures are likely to impede their career control and adaptability (Zhang and Wei, 2018). This could explain the higher probability of female teachers falling into the relatively lower adaptability group, which reflects a lack of career adaptability across multiple dimensions.

Age factors (including age and teaching year) did not increase/decrease the probability of falling into the moderate adaptability with stronger control or the high adaptability. Although previous research has found that teachers’ career adaptability shows significant differences in teaching age (Zhang and Wei, 2018), Rudolph et al. (2017) meta-analysis results indicate a weak correlation between age and career adaptability. The possible explanation for the results is the level of career adaptability is not solely dependent on individual’s age factors but is also influenced by other variables. Overall, some age-related factors may contribute to career adaptability, such as increased responsibility and agreeableness with age, while other factors may decrease it, such as decreased cognitive ability and flexibility with age, and these age-related changes may offset each other in general, resulting in a weaker overall correlation between age factors and career adaptability.

Moreover, being normal teachers who learn education did not raise the likelihood of belonging to the high adaptability group, whereas being non-teaching major teachers was associated with a higher probability of falling into the high adaptability category. This is possible because non-teaching major teachers likely bring broader skill sets and perspectives to their teaching roles, allowing them to navigate career challenges more effectively. This aligns with prior findings that career adaptability is influenced by exposure to diverse experiences and opportunities, which can foster career curiosity, confidence, and control (Meng and Briscioli, 2024). Additionally, educational level and professional title did not predict differences in profile membership between the relatively lower adaptability and the moderate adaptability with stronger control or the high adaptability. This suggests that these factors may not be strong determinants of career adaptability in the Chinese teaching context. One possible explanation is that career adaptability may be shaped more by psychological factors, such as personality traits and contextual factors like social support, rather than solely by demographic characteristics. These results underscore the significance of incorporating a broader range of factors when studying career adaptability and imply that demographic variables may not consistently serve as reliable predictors of career adaptability outcomes.

#### 5.2.2 The role of big five traits in career adaptability profiles

To explore the potential predictors behind the different profiles, this study also investigated the relationship between Big Five traits and adaptability profiles, in addition to examining demographic variables. With Hypothesis 3, the present study expected teachers’ low level of negative emotionality and high levels of extraversion, agreeableness, conscientiousness, and open-mindedness will contribute to high career adaptability. This hypothesis received partial support. As expected, the findings revealed that teachers with lower negative emotionality (neuroticism) levels and high levels of extraversion, conscientiousness, and open-mindedness had a higher probability of falling into the high adaptability profile and a reduced likelihood of being classified into the relatively lower adaptability or moderate adaptability with stronger control profiles. This finding conceptually aligns with prior research showing that nursing students in the adaptive ready profile, which was characterized by low neuroticism and high mean levels of other four traits, perceived a higher level of career adaptability, organized study behaviors, and academic satisfaction (Perera and McIlveen, 2017). This similar pattern suggests that low neuroticism combined with higher levels of other Big Five traits may represent an adaptive readiness pattern associated with stronger career adaptability. Additionally, workers with lower neuroticism levels had higher chances of falling into sustainable types of career trajectories, thus avoiding unsustainable career (Udayar et al., 2024).

Unexpectedly, teachers who scored higher on agreeableness were found to be more inclined to exhibit moderate adaptability with stronger control profile. This is not entirely surprising, as the nuanced effects of agreeableness in the workplace have been discussed previously. Research has found that workers with higher levels of agreeableness are more likely to encounter unsustainable career trajectories (Udayar et al., 2024). Importantly, this result does not necessarily conflict with the positive role of conscientiousness. Although agreeableness and conscientiousness are both socially valued traits, conscientiousness is more closely related to goal-directed self-regulation, persistence, and achievement-oriented engagement, which may explain why it was associated with the high adaptability profile. By contrast, agreeableness is more closely related to interpersonal harmony, conflict avoidance, and compliance with expectations. The unexpected result in the present study might be due to the special cultural and professional background in China. In collectivist societies such as China, agreeableness is more a function of the desire to maintain harmony, avoid conflict, and comply with social norms. Although these traits are highly valued, they may not necessarily translate into the highest level of career adaptability, as such workers may focus more on meeting institutional needs than on personal career exploration. This tendency may be particularly pronounced in the field of education, a field characterized by stability, admiration and great pressure to appease societal expectations. Teachers high in agreeableness tend to comply with established norms and prioritize the needs of students, parents, and administrators, neglecting their own professional development and involvement in career adaptability tasks such as attempting to broaden or acquire new professional roles and develop new skills. Furthermore, increased social desirability may drive these teachers to maintain an image of compliance and dedication, further reinforcing a repressed approach to career adaptability and inhibiting internal motivations for change or development.

### 5.3 Outcomes of career adaptability profiles

The analysis of mental health differences between profiles also provided valuable insights. In line with our hypotheses, mental health was evaluated across all indicators, revealing significant differences across profiles. Profiles exhibiting higher career adaptability consistently showed better mental health outcomes. These results are consistent with previous prior variable-centered research, which highlighted that higher levels of teacher career adaptability are associated with improved mental health (Shava and Chinyamurindi, 2021). Additionally, it is worth noting that primary and secondary school teachers in the relatively lower adaptability profile exhibited the lowest levels of mental health, indicating that a significant proportion of teachers still face concerning mental health challenges. Research on the changing mental health trends of primary and secondary school teachers in China has revealed a steady increase in psychological issues over the years, particularly in the dimensions of hostility and psychoticism (Yang et al., 2019). The above-mentioned issues are closely related to social relationship, occupational stress and psychological resources (Hascher and Waber, 2021; Huang et al., 2024). These factors share similarities with the dimensions of career adaptability (Shava and Chinyamurindi, 2021). Career adaptability reflects the fit between individuals and their career environments, while the goodness of mental health is usually addressed as a career adaptation result (Aminah et al., 2023; Rudolph et al., 2017). When there are insufficient adaptability resources for primary and secondary school teachers to counterbalance their occupation or suffer from poor mental health or even show depression symptoms, students may also be vulnerable to these forms of psychological status in such conditions (Yan et al., 2024). This result highlights the urgency for educational authorities and schools to pay more attention to the mental health of teachers from the relatively lower adaptability cluster.

Teachers in the moderate adaptability with stronger control group demonstrated moderate levels of mental health, consistently superior to those in the relatively lower adaptability group but lower than those with the high adaptability profile. This middle point is considered to be a degree of career adaptability that offers some protection against stress, yet it is not enough to ensure optimal well-being. While these teachers have fundamental coping skills, they do not have proactive and reflective strategies to thrive in dynamic work settings. The “repressed” element suggests an internal conflict: these teachers may recognize the need for change but feel constrained by external circumstances or internal barriers such as fear of failure. This state is consistent with the action–state orientation perspective stressing the negative effect of low initiative on mental health (Birk et al., 2020).

Overall, the present study underscores the importance of career adaptability in impacting the mental health outcomes of teachers at the primary and secondary school levels. By characterizing unique profiles of adaptability and the subsequent psychological consequences, this study lays the groundwork for interventions that focus on developing career adaptability as a means of creating mental health among teachers. In the future, these relationships need to be tested in diverse samples and contexts, in order to continue strengthening our knowledge of the relation between career adaptability and mental health.

### 5.4 Implications

This study provides tentative implications for teacher career adaptation and development. The personal-centered approach to investigating teacher career adaptability offers a robust framework for identifying latent subgroups of primary and secondary school teachers with distinct intra-individual patterns of career adaptability based on the assumption that the sample comprises a mix of heterogeneous subpopulations. Accordingly, this study contributes theoretically and methodologically to the CCT in demonstrating how career adaptability can be adequately captured through latent profiles representing distinct patterns of adaptability resource configurations. These profiles provide nuanced insights as to how primary and secondary school teachers use career concern, control, curiosity and confidence to manage career-related adversity and sustain mental health.

From a career counseling practice perspective, the identification of unique career adaptability profiles of Chinese primary and secondary school teachers challenges a “one-size-fits-all” approach of teacher professional development. Instead, interventions that correspond with specific career adaptability resource profiles should be suggested. For example, the findings indicate that it could be feasible for educators and career counselors to identify groups of teachers with a configuration of low career adaptability (i.e., relatively lower adaptability). Teachers in these groups are more likely to possess fewer adaptability resources to cope with academic and career-related challenges and, thus, may increase the risk of experiencing poor mental health. Structured interventions aimed at ameliorating the uneven development of these teachers’ adaptability resources by boosting their adaptivity (i.e., personality traits) may better equip them to navigate new academic tasks and professional challenges, achieving optimal adaptation. Tailored interventions at the level of latent profiles are likely to be more economically sustainable and precise than generalized training programs, especially when integrated into mandatory professional development systems on a large scale.

### 5.5 Limitations and directions for future research

This study also has some limitations that serve to qualify the findings and provide directions for future work. First, the use of a cross-sectional design limits the capacity to make causal inferences about the relationships between the antecedents and outcomes of career adaptability profiles. Future investigations should employ longitudinal designs for a more detailed investigation of the temporal sequencing of these constructs and improve the construct validity and the replicability of career adaptability profiles.

The second limitation concerns the generalizability of the identified profiles. Although results are indicative of the replicability of career adaptability profiles among primary and secondary school teachers, they should be considered a first step in identifying wider generalization capabilities. The present sample was recruited through convenience sampling from Guangzhou, one of China’s more economically developed urban areas. Therefore, the findings should not be generalized directly to all Chinese primary and secondary school teachers, particularly those working in rural, inland, or less economically developed regions, who may face different career pressures, institutional resources, and professional development opportunities. To further advance teacher career adaptability theory more research should probe whether these profiles are consistent across (a) diverse groups defined by sociodemographic characteristics (for example, region, cultural context) and (b) educational systems, institutional settings (e.g., public vs. private) and job roles (e.g., general classroom teachers vs. specialist subject teachers). Such variations investigation will yield valuable insights into the contextual and individual factors influencing career adaptability and would both the theoretical and practical applications of this framework.

Another limitation concerns the age distribution of the sample. Although participants ranged in age from 20 to 44 years, the sample was relatively young overall, with a mean age of 26.33 years. Thus, mid- and late- career teachers may have been underrepresented. This restricted age range may partly explain the nonsignificant effects of age and teaching experience found in this study. Therefore, the null age-related findings should be interpreted cautiously and should not be taken as evidence that career adaptability profiles are unrelated to age or career stage. Future research should include teachers across a broader range of ages, teaching experience, and career stages to examine whether career adaptability profiles differ among early-, mid-, and late-career teachers.

A further limitation concerns the measurement structure of career adaptability. The present study used the 24-item CAAS-China Form and did not separate a general career adaptability component from dimension-specific variance. Given the high correlations among the four CAAS dimensions, the predominantly level-based profile pattern may partly reflect shared variance among concern, control, curiosity, and confidence rather than only substantive profile heterogeneity. Future research could use bifactor models or residualized profile indicators to examine whether more qualitatively distinct profiles emerge after accounting for shared variance among the CAAS dimensions, and should also consider recent evidence on differences across CAAS forms and reliability estimates (Song et al., 2025; Xu et al., 2025).

## 6 Conclusion

This study advanced research on teachers’ career adaptability by adopting a personal-centered approach and using a prospective cross-sectional design to identify distinct profiles of career adaptability. We also examine the relationships between profile membership and sociodemographic characteristics, the Big Five personality dimensions (adaptivity), and mental health (adaptation) within the framework of the CCT perspective. Latent profile analyses showed that a comparable 3-profile solution varied between types of career adaptability profiles. The analysis demonstrated that most of the Big Five traits were associated with higher career adaptability level, and teachers with non-teaching majors were more likely to belong to the high adaptability profile. Inversely, the agreeableness trait predicted cross-sectionally membership only in the moderate adaptability with stronger control profile, rather than in the high adaptability profile. Additionally, teachers in the relatively lower adaptability profile reported systematically lower levels of mental health than teachers in the moderate adaptability with stronger control or high adaptability profiles. The findings indicate that the three profiles are interrelated and when career-related adversities and adverse workplace incidents occur, they influence general mental health as significantly as they affect occupation-specific performance.

## Data Availability

The original contributions presented in the study are included in the article/supplementary material, further inquiries can be directed to the corresponding author.
